# On the Properties of British Opium

**Published:** 1828-06

**Authors:** 


### BRITISH OPIUM.

### *On the Properties of British Opium.* By a Member of the College of Surgeons.

Having been in the habit for several years of using British opium in my practice, and being satisfied of its great superiority to the Turkey opium in ordinary use, I beg leave, through the medium of your widely circulated Journal, to call the attention of the profession to the following facts respecting it.

It is superior in point of purity; the British being free from that admixture of foreign substances which are always found even in the best specimens of the Turkey.

Its *anodyne* properties are quite equal to those of the best Turkey, while its *narcotic* or deleterious properties are much weaker. Thus, I find one grain of the substance, or fifteen minims of the tincture, a sufficient dose in ordinary cases; but herein its superiority is manifest, that when the urgency of symptoms requires it, three or four grains may be given without much risk of its producing the deleterious effects which would be almost certain to follow the same dose of Turkey opium. I have given it to the extent of from three to six grains in the day in a great number of cases, and I only recollect two in which it had any unpleasant effect, while in most instances it has produced decided relief of pain, without exciting the least headache, vertigo, or confusion of intellect. A friend of mine, who is in the habit of taking one or two grains of opium occasionally at bedtime, complained to me of the headache which he always experienced the next day, particularly if any thing occurred to disturb his rest after a dose. I gave him a few pills of the British opium: he was astonished at the effect of them, declaring that they gave him comfortable rest, without producing any thing like the degree of headache which he had always experienced before.

It would be gratifying to me, and useful to the profession, if Mr. Battley, or one of your chemical correspondents, would ascertain whether this effect of the British opium arises (as I suspect it does) from its containing a larger proportion of morphium, and a less proportion of narcotine than the Turkey. Be this as it may, I was gratified a short time ago to observe, from a report of the French Institute, that they had come to the same conclusion respecting opium grown in France, as I had done respecting the British.

I could cite many cases in confirmation of the opinion I have advanced, but I hope I have said enough to induce medical men to give it a trial. I fear, indeed, that the present low price of Turkey opium will tend to prevent the cultivation of the British; but I am persuaded, if the profession were fully aware of the superior virtues of the latter, they would prefer it even at some advance of price. I consider, indeed, that its value may be stated to be at least one-third more than that of the best specimens of Turkey. As it is not always to be obtained in the market, I will add for the information of such of your readers as may wish to try it, that I procured it at Messrs. Winstanley and Son's, in the Poultry.

